# Recombinant human thrombomodulin inhibits neutrophil extracellular trap formation in vitro

**DOI:** 10.1186/s40560-016-0177-9

**Published:** 2016-07-22

**Authors:** Yasuyo Shimomura, Mika Suga, Naohide Kuriyama, Tomoyuki Nakamura, Toshikazu Sakai, Yu Kato, Yoshitaka Hara, Chizuru Yamashita, Hiroshi Nagasaki, Masao Kaneki, Osamu Nishida

**Affiliations:** Department of Anesthesiology and Critical Care Medicine, Fujita Health University School of Medicine, 1-98 Dengakugakubo, Kutsukake-cho, Toyoake, Aichi 470-1192 Japan; Department of Physiology I, Fujita Health University School of Medicine, 1-98 Dengakugakubo, Kutsukake-cho, Toyoake, 470-1192 Japan; Department of Anesthesia, Critical Care and Pain Medicine, Massachusetts General Hospital, Harvard Medical School, 149 13th Street, Charlestown, MA 02129 USA

**Keywords:** Disseminated intravascular coagulation, Innate immunity, Neutrophil extracellular traps, Sepsis, Thrombomodulin

## Abstract

The aim of this study was to investigate the effects of recombinant human-soluble thrombomodulin (rTM) on lipopolysaccharide (LPS)-induced, platelet-dependent neutrophil extracellular trap (NET) formation (NETosis). Human peripheral blood neutrophils and platelets were co-incubated with or without LPS (0.2 μg/ml) in the presence and absence of rTM (2 μg/ml). NETosis was confirmed by immunostaining and confocal microscopy. In the absence of platelets, LPS did not induce NETosis in the neutrophils. NETosis, however, was induced by LPS when neutrophils were co-cultured with platelets (64 % of neutrophils). Notably, rTM was able to fully inhibit NETosis in neutrophils cultured with platelets and in the presence of LPS. rTM did not induce NETosis in this co-culture system (*p* < 0.01 versus LPS in the absence of rTM). These results show that rTM can suppress LPS-induced platelet-dependent NETosis in vitro.

## Introduction

Neutrophil extracellular traps (NETs) are important for local defense against invading pathogens [1]. However, NET formation, also known as NETosis, can cause or exacerbate hypercoagulability, microvascular thrombosis, and vascular endothelial dysfunction in critical illness, including sepsis [2].

Thrombomodulin (TM) is mainly expressed on the cell surface of endothelial cells, but the soluble form of TM also exists in the circulation [3]. TM exerts protective effects against endothelial dysfunction and damage associated with sepsis and coagulopathy [4]. However, the mechanisms underlying the protective effects of TM in endothelial cells are not completely understood. Recently, recombinant human-soluble TM (rTM) has been used for the treatment of patients with sepsis-associated disseminated intravascular coagulation (DIC) in Japan, and rTM treatment has shown protective effects [5].

A previous study demonstrated that lipopolysaccharide (LPS) induces NET formation in a platelet-dependent manner [2]. Consistent with this previous study, we found that while neither LPS nor platelets alone caused NETosis in cultured neutrophils, LPS co-cultured with neutrophils and platelets markedly induced NETosis. These results clearly indicate that LPS-induced NET formation is platelet-dependent.

We noticed that NET formation induced by both LPS and activated platelets resembled a serious condition of inflammation in response to infection, such as during septic shock and sepsis-associated DIC. However, the effects of rTM on NET formation have not been studied. Therefore, in this study, we investigated the effects of rTM on NETosis in the presence of both LPS and platelets in vitro.

## Methods

### Isolation of human neutrophils and platelets

Peripheral blood was collected in EDTA-containing Vacutainers from ten healthy volunteers. Neutrophils were isolated using Polymorphprep (Axis-Shield, Dundee, UK). For platelet isolation, 2 ml of whole blood containing 3.2 % sodium citrate solution was centrifuged at 300 *g* for 5 min at 4 °C. Platelet-rich plasma was centrifuged at 1000 *g* for 15 min at 4 °C, the supernatants were removed, and the cell pellets were resuspended in PBS.

### In vitro NET formation

In vitro NETosis was induced as previously described [6] with minor modifications. In brief, neutrophils (5 × 10^4^) were incubated in PBS containing 10 % fetal bovine serum with or without platelets (5 × 10^5^). LPS (*Escherichia coli* 0111, 125-05201, Wako Pure Chemical Industries, Ltd., Osaka, Japan) was added at 0.2 μg/ml to stimulate cells. To investigate the effect of rTM on the inhibition of NETosis, incubated cells were treated with rTM (2, 10, or 50 μg/ml (39, 192, or 960 nM), ART-123, provided by Asahi Kasei Pharma Corp., Tokyo, Japan) immediately after LPS stimulation and incubated for 30 min at 37 °C in 5 % CO_2_. rTM concentrations were chosen based on previous studies [7, 8]. Clinically, rTM (0.06 mg/kg) was administered intravenously once daily for six consecutive days, and Cmax (maximum plasma drug concentration) reached 1600 ng/ml [7]. Furthermore, previous studies used rTM concentrations of 2 or 20 μg/ml in vitro [8]. Therefore, rTM was added at the concentrations of 2, 10, or 50 μg/ml in this study.

### Immunostaining and confocal microscopy

Cells were stained with an anti-myeloperoxidase (MPO) (ab45977, Abcam, Cambridge, MA, USA) or histone H2A.X antibody (sc-54607, Santa Cruz Biotechnology, Santa Cruz, CA, USA), followed by species-specific secondary antibodies conjugated with Alexa Fluor Dyes (Invitrogen, Life Technologies Japan, Tokyo, Japan). DNA was stained with DAPI (D1306, Invitrogen, Life Technologies). NETosis was evaluated by the release of MPO, histone H2A.X, and DNA from the neutrophils using a confocal microscope (LSM 710 confocal microscope; Carl Zeiss MicroImaging, Jena, Germany). The numbers of NETs and total neutrophils were counted, and the percentage of NETs in the total neutrophils was calculated as previously described [9, 10].

### Statistical analyses

The percentage of NETs was expressed as median with interquartile range (IQR) and compared using the Kruskal-Wallis one-way analysis of variance on ranks.

## Results

The number of neutrophils was counted in the observation view field of a microscope image.

**Fig. 1:**
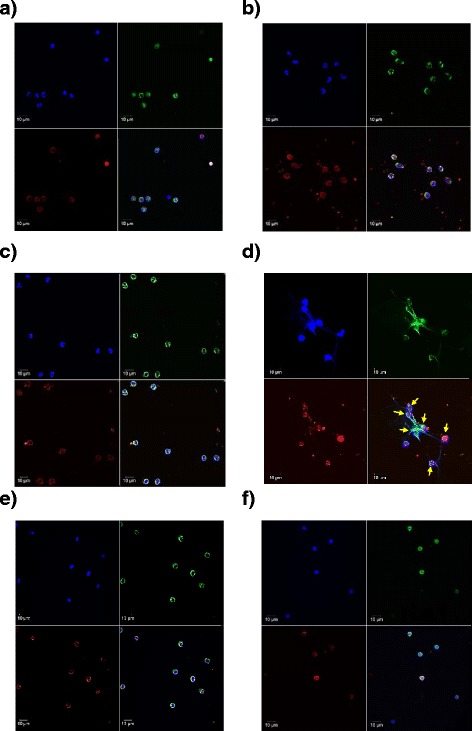
Inhibition of LPS-stimulated platelet-dependent NETosis by rTM. Neutrophils (5 × 10^4^) and platelets (5 × 10^5^) from the same individuals were resuspended in 100 μl of PBS containing 10 % fetal bovine serum. Cultured cells (neutrophils alone or with platelets) were incubated with or without LPS (0.2 μg/ml) in the presence or absence of rTM (2 μg/ml). Immunostaining for MPO and histone H2A.X was performed, and nuclei were stained with DAPI. **a–d** Normal neutrophils were cultures alone **(a)**, with LPS alone **(b)**, with platelets alone **(c)**, or co-cultured with platelets and LPS **(d)**. **e**–**f** rTM was added to the co-culture of neutrophils and platelets with **(e)** or without LPS **(f)**. *Arrows* indicate neutrophils that underwent NETosis. *Blue*, DAPI; *green*, MPO; *red*, histone H2A.X; *yellow*, merged signals. Magnification: ×63, scale bar = 10 μm

LPS did not induce NETosis when neutrophils were not co-cultured with platelets. Without LPS stimulation, NETosis did not occur in the co-culture of neutrophils and platelets (Fig. 1a–c). However, when neutrophils cultured in the presence of platelets were stimulated by LPS, neutrophils released chromatin (observed by histone H2A.X staining) lined with granular components (observed by MPO staining), creating fibrous nets in the cytoplasm. These observations confirmed that neutrophils emitted NETs (Fig. 1d). This confirms previous observations showing that the NETosis induced by LSP is platelet-dependent [2].

To investigate the effect of rTM on LPS-induced platelet-dependent NETosis, rTM was added to the co-culture of neutrophils and platelets. In the presence of rTM (2, 10, or 50 μg/ml), LPS failed to induce NETosis. Our results showed that rTM at 2 μg/ml was sufficient to block LPS-induced NETosis (Fig. 1e). The results showed that rTM inhibited the LPS-induced platelet-dependent NETosis, as indicated by the unchanged intracellular localization of DNA, MPO, and H2A.X (Fig. 1e). On the other hand, rTM did not induce NETosis in the absence of LPS (Fig. 1f).

**Fig. 2:**
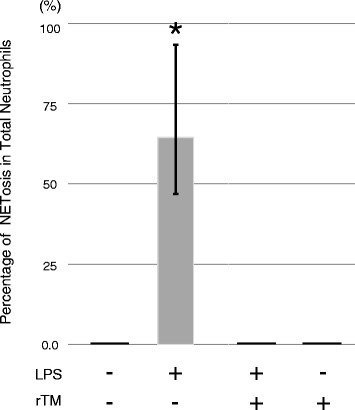
Quantification of inhibition of LPS-stimulated platelet-dependent NETosis by rTM. Neutrophils and platelets were isolated from 11 healthy adult volunteers and treated and stained as in Fig. 1. Quantification of NETosis was performed by counting the numbers of neutrophils that underwent NETosis and those of total neutrophils in the co-culture of neutrophils and platelets. Neutrophil and platelet treatment groups were control/untreated (*n* = 10), LPS alone (*n* = 10), LPS in the presence of rTM (*n* = 10), and rTM alone (*n* = 3). Data are shown as the median with interquartile range. Kruskal-Wallis one-way analysis of variance on ranks (*p < 0.01 versus control and rTM treatment, *n* = 11 individuals)

Quantification of NETosis in the co-culture of neutrophils and platelets revealed that the addition of LPS to the neutrophil-platelet co-culture induced NETosis in 64 % of neutrophils (IQR, 36–82 %) (Fig. 2). However, rTM at 2 μg/ml was sufficient to block LPS-induced NETosis to 0 % (IQR, 0.0–0.03 %]) (*p* < 0.01 versus LPS alone) (Fig. 2). Together, our findings demonstrate that rTM is capable of suppressing NETosis in vitro.

## Discussion

Our results showed that LPS stimulation did not induce NETosis from neutrophils without co-culture with platelets. These results are consistent with findings from a previous study [2]. Importantly, our study revealed that LPS-induced platelet-dependent NETosis was inhibited by rTM.

NETosis in the microvasculature is an important molecular event causing endothelial dysfunction [2]. Our data raise the possibility that endogenous TM both in the endothelial cells and in the circulation (the soluble form of TM) may decrease NETosis and thereby prevent endothelial dysfunction contributing to the improvements in the clinical outcome of patients with sepsis-associated DIC.

Yipp et al. described two types of NETosis that occur via two distinct pathways [11]. Phorbol 12-myristate 13-acetate-induced NETosis is associated with nicotinamide adenine dinucleotide phosphate oxidase-dependent cellular death. This pathway requires hours for release of NETs. In contrast, microbial exposure or LPS-induced NETosis occurs rapidly (<30 min) [2, 12]. Moreover, the direct interaction of activated platelets with neutrophils causes NETosis, particularly when platelets are activated via Toll-like receptor (TLR) 4-mediated signaling [2]. Coagulopathy is an integral component of the development of severe sepsis. To investigate the effects of rTM on NETosis, we therefore used the LPS-stimulated platelet-dependent NETosis, a clinically relevant model of NETs in the context of coagulopathy and sepsis.

As a limitation of these in vitro experiments, however, the mechanisms of the rTM-mediated inhibition of NETosis are not clear and remain to be determined. It is possible that rTM may exert an inhibitory effect against TLR4-mediated signaling. We are currently attempting to clarify the mechanisms by which rTM inhibits LPS-induced NETosis.

Our findings suggest that rTM and possibly endogenous TM may have a novel role in the regulation of NETosis. These data raise the possibility that the inhibition of NETosis may contribute to the protective effects of rTM in patients with sepsis and/or DIC. Further studies are required to determine whether rTM inhibits NETosis in vivo in patients with sepsis and/or DIC.

## Abbreviations

Cmax, maximum plasma drug concentration; DIC, disseminated intravascular coagulation; IQR, interquartile range; LPS, lipopolysaccharide; MPO, myeloperoxidase; NETs, neutrophil extracellular traps; rTM, recombinant human-soluble thrombomodulin; TLR, Toll-like receptor
